# Huntingtin reduction results in altered nuclear structure and heterochromatic instability

**DOI:** 10.1093/hmg/ddaf126

**Published:** 2025-08-01

**Authors:** Jessica C Barron, Sean T Coady, Abigayle C Fleming, Samantha J Carew, Makenna C A Taylor, Emily P Hurley, Firoozeh Nafar, Matthew P Parsons

**Affiliations:** Division of Biomedical Sciences, Faculty of Medicine, Memorial University, 300 Prince Philip Drive, St. John’s, NL, A1B 3V6, Canada; Division of Biomedical Sciences, Faculty of Medicine, Memorial University, 300 Prince Philip Drive, St. John’s, NL, A1B 3V6, Canada; Division of Biomedical Sciences, Faculty of Medicine, Memorial University, 300 Prince Philip Drive, St. John’s, NL, A1B 3V6, Canada; Division of Biomedical Sciences, Faculty of Medicine, Memorial University, 300 Prince Philip Drive, St. John’s, NL, A1B 3V6, Canada; Division of Biomedical Sciences, Faculty of Medicine, Memorial University, 300 Prince Philip Drive, St. John’s, NL, A1B 3V6, Canada; Division of Biomedical Sciences, Faculty of Medicine, Memorial University, 300 Prince Philip Drive, St. John’s, NL, A1B 3V6, Canada; Division of Biomedical Sciences, Faculty of Medicine, Memorial University, 300 Prince Philip Drive, St. John’s, NL, A1B 3V6, Canada; Division of Biomedical Sciences, Faculty of Medicine, Memorial University, 300 Prince Philip Drive, St. John’s, NL, A1B 3V6, Canada

**Keywords:** Huntingtin, Huntington’s disease, Chromatin, Transcription, Epigenetics

## Abstract

Huntington’s disease (HD), a fatal neurodegenerative disease, arises due to a CAG repeat expansion in the huntingtin (*HTT*) gene. Non-pathogenic wild type HTT (wtHTT) is essential for neurodevelopment as well as many vital cellular functions within the adult brain; however, the consequences of wtHTT reduction in adulthood and particularly in extrastriatal regions of the brain have not been well characterized. Understanding the implications of wtHTT loss is essential as numerous genetic therapies for HD non-specifically reduce the expression levels of both mutant and wtHTT. The aim of the current study was to characterize the effect of wtHTT reduction from the whole cell to synaptic level in primary hippocampal neurons using conventional and super-resolution imaging methods. Our results identified the nucleus as an organelle that is particularly vulnerable to wtHTT reduction, with hippocampal neurons exhibiting increased nuclear size relative to the soma, DNA decompaction and a progressive loss of heterochromatin, and biphasic changes in nuclear pCREB signaling following siRNA-mediated wtHTT knockdown. Other structural assessments including dendritic complexity, spine density and synaptic morphology appeared to be largely unaffected in our wtHTT-lowered cells. These findings highlight the nucleus as an organelle that may be particularly sensitive to huntingtin-lowering in the mammalian brain.

## Introduction

An expanded CAG repeat in the huntingtin gene (*HTT*) causes Huntington’s disease (HD), a fatal neurodegenerative disease for which there is currently no cure. Various HTT-lowering therapeutics have entered clinic trials in recent years; however, many of these drugs are non-selective and lower protein levels of both mutant HTT (mHTT) and wild-type HTT (wtHTT) [1]. A prominent non-selective antisense oligonucleotide-based HD therapeutic tominersen was abruptly halted in March 2021 in phase III of its human clinical trial. During this trial, HD patients receiving tominersen every 8 weeks showed significantly worse clinical outcomes compared to patients given placebo treatments and patients given the drug every 16 weeks showed no clinically detectable improvements [2]. wtHTT is essential for neurodevelopment, as constitutive knockout (KO) of *Htt* in mice is embryonic lethal at E8-E9 [3–5]. Additionally, wtHTT has been identified as an essential hub protein in the brain. In 2012, Shirasaki *et al.* identified 747 putative proteins within the HTT interactome [6]. More recent proteomic studies further emphasize the intricate complexity of the HTT interactome, with abundant direct and indirect protein interactions being influenced by polyglutamine length [7, 8]. As HD patients entering HTT-lowering clinical trials have already reached adulthood, understanding the potential consequences of reducing wtHTT in the mature brain is critical for the development of safe and effective HD therapeutics.

wtHTT is a major scaffolding protein that influences the axonal transport of essential cargos within neurons. These include RNA granules containing mRNA, such as β-actin mRNA which is known to regulate dendritic and spine morphology [9, 10]. Loss of wtHTT disrupts the bidirectional transport of the essential neurotrophin BDNF as well as its receptor TrkB [11–13]. At the synapse, the BDNF–TrkB pathway is essential for long-term potentiation (LTP), and the healthy transport of TrkB-containing vesicles can enhance dendritic complexity and exert pro-survival effects [14]. We have recently reviewed the role of wtHTT at the synapse and suggested it is likely to play a critical role in synaptic homeostasis through its various interactions with protein partners that regulate synaptic neurotransmission and plasticity [15]. Indeed, our group has recently demonstrated that wtHTT is essential for long-term synaptic plasticity in the adult mammalian hippocampus [16]. Synapse-to-nuclear transport of numerous transcription factors, including NF-κB, is also positively regulated by wtHTT. NF-κB relies on the retrograde motor proteins dynein/dynactin for its transport to the nucleus [17], and wtHTT supports the structural integrity of the dynein/dynactin complex [18] which is disrupted by mHTT [19]. wtHTT can also influence transcription in myriad ways, for example through interactions with a variety of transcription factors, activators, and repressors, including CREB binding protein (CBP), NF-κB, p53, and REST/NRSF, among many others [20, 21]. As synaptic dysfunction and transcriptional dysregulation are both observed in HD [22, 23], and given the non-selective nature of numerous huntingtin-lowering therapeutics, it is imperative that we increase our understanding of how wtHTT reduction may negatively impact the fundamental properties of mature brain cells.

The aim of the current study was to provide a thorough characterization of the effect of wtHTT reduction on hippocampal neurons. Results presented here indicate that wtHTT-lowered neurons have an increased nuclear size relative to the soma, with nuclei presenting in a more relaxed, euchromatic state. We further found that wtHTT loss produced a time-dependent biphasic effect on the transcription factor CREB, with an initial reduction of nuclear pCREB followed by a marked increase at later stages of knockdown. The shift in pCREB was accompanied by a progressive decrease in the repressive histone marker H3K9me3, suggesting chromatin relaxation over time. Together, these findings point to the nucleus as an early and evolving site of dysregulation in response to wtHTT reduction.

## Results

### wtHTT reduction in mixed neuron-astrocyte hippocampal cultures

**Figure 1:**
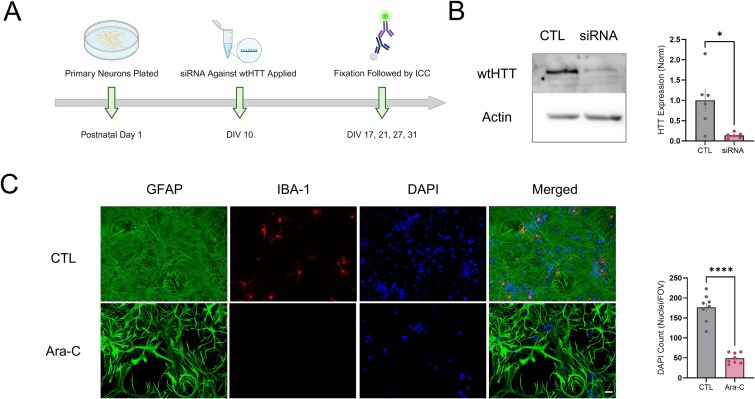
(A) schematic of primary hippocampal culture treatment protocol created using BioRender. (B) left: Total protein from DIV 17 primary hippocampal neurons was collected and wtHTT levels were assessed using western blot. Right: Normalized wtHTT protein levels relative to actin control values. (C) left: Representative GFAP, IBA-1 and DAPI stained cells with or without Ara-C treatment to show mixed neuron and astrocyte culture composition. Scale bar represents 20 μm. Right: Analysis of nuclei per field of view (FOV). Data were assessed by unpaired t-tests and are represented as mean ± SEM. ^*^P < 0.05, ^*^^*^^*^^*^P < 0.0001.

wtHTT levels were reduced in primary hippocampal neurons by siRNA application at DIV 10. To assess increasing durations of wtHTT reduction, cells were PFA fixed at either DIV 17, DIV 21, DIV 27, or DIV 31 followed by various ICC staining protocols (Fig. 1A). To validate wtHTT protein reduction in our primary cultures, we collected protein at DIV 17, one week after siRNA treatment, and assessed wtHTT levels using Western blot. At DIV 17, wtHTT siRNA-treated cells showed an 86.3% reduction in wtHTT compared to the scrambled control siRNA (Fig. 1B, *P* = 0.012). We also confirmed siRNA efficacy at DIV 31 where we saw a similar reduction in wtHTT at this later timepoint (Fig. S2A and B). To inhibit glial proliferation and reduce overall density, primary cultured cells were treated with Ara-C starting at DIV 2. In order to assess culture composition with or without Ara-C, control and Ara-C treated cells were fixed at DIV 17 and stained for GFAP and IBA1 to visualize astrocytes and microglia, respectively. As expected, Ara-C reduced astrocytic growth and completely eliminated microglia, leading to an overall decrease in cell density as assessed by DAPI nuclear count (Fig. 1C, *P* <  0.0001). Ara-C treated neurons also appeared to grow and mature in a healthy manner (Fig. S3A). To further gauge cell health in our cultures following wtHTT knockdown, cultured hippocampal cells were stained with nuclear marker DAPI, and were imaged using conventional widefield microscopy (Fig. S4A). We quantified nuclear size and density to determine whether significant nuclear condensation or cell loss was occurring following siRNA treatment. We found that wtHTT reduction had minimal effect on the average nuclear size (Fig. S4B, DIV 17 *P* = 0.258, DIV 21 *P* = 0.004; DIV 27 *P* = 0.281; DIV 31 *P* = 0.844) and no effect on cell density (Fig. S4C; DIV 17 *P* = 0.929, DIV 21 *P* = 0.845; DIV 27 *P* = 0.227; DIV 31 *P* = 0.629). At DIV 21 we did observe a significant decrease in average nuclear size in the siRNA-treated cells, though this effect was not observed at any other time point. Thus, the wtHTT siRNA used in the present study successfully lowers wtHTT expression and appears not to induce any considerable amount of cell death in these cultures.

### Super-resolution analysis of synaptic morphology of wtHTT-lowered primary hippocampal neurons

**Figure 2:**
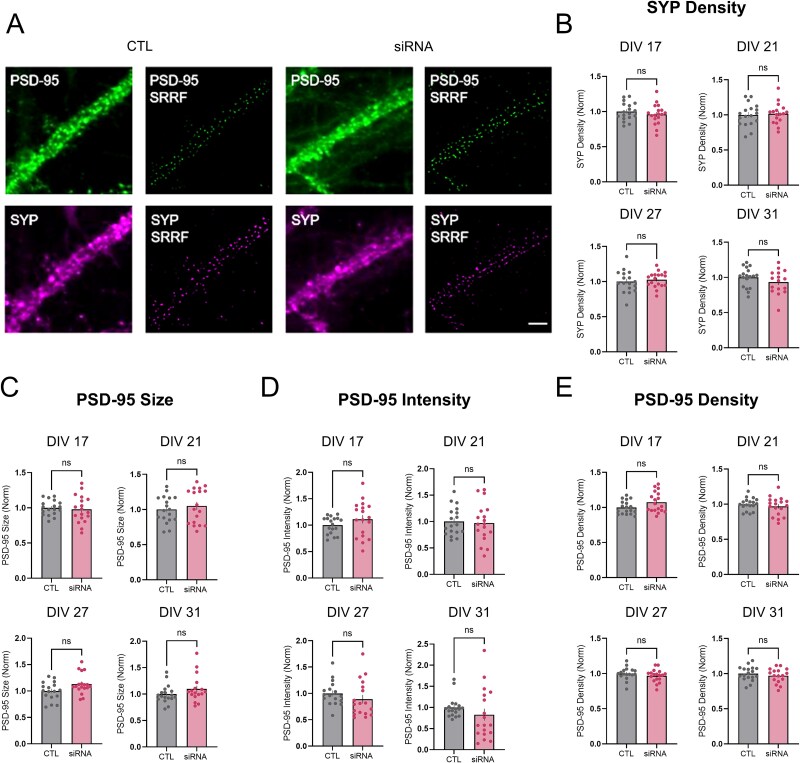
(A) dendritic segments before and after super-resolution radial fluctuations (SRRF) processing to visualize postsynaptic and presynaptic sites, PSD-95 and synaptophysin (SYP), respectively. Scale bar represents 5 μm. (B) analysis of SYP puncta density values. (C) analysis of PSD-95 puncta size values. D analysis of PSD-95 puncta intensity values. E analysis of PSD-95 puncta density values. Data were assessed by unpaired t-tests and Mann–Whitney tests and are represented as mean ± SEM. Ns: Nonsignificant.

Due to the association of wtHTT with numerous proteins required for healthy synaptic structure and function [15], we investigated the consequences of wtHTT reduction on synaptic morphology in primary hippocampal culture. To do this, we incorporated the super-resolution method SRRF in a similar manner as previously demonstrated by our lab [24]. Presynaptic terminals were ICC labelled with an antibody against the synaptic vesicle protein synaptophysin (SYP), and excitatory postsynaptic sites were immunostained using an antibody against the postsynaptic density scaffold protein PSD-95. SRRF processing drastically improved the resolution of our images of synaptic protein puncta (Fig. 2A). Overall, wtHTT reduction had no significant effect on the properties of PSD-95 or SYP puncta (Fig. 2B–E). More specifically, siRNA treatment had no significant effect on SYP puncta density (Fig. 2B, DIV 17 *P* = 0.380; DIV 21 *P* = 0.743; DIV 27 *P* = 0.554; DIV 31 *P* = 0.222), PSD-95 puncta size (Fig. 2C, DIV 17 *P* = 0.694; DIV 21 *P* = 0.542; DIV 27 *P* = 0.072; DIV 31 *P* = 0.163), PSD-95 puncta intensity (Fig. 2D, DIV 17 *P* = 0.210; DIV 21 *P* = 0.803; DIV 27 *P* = 0.126; DIV 31 *P* = 0.079) or PSD-95 puncta density (Fig. 2E, DIV 17 *P* = 0.074; DIV 21 *P* = 0.426; DIV 27 *P* = 0.293; DIV 31 *P* = 0.402). These data suggest that wtHTT reduction under the present experimental conditions has no effect on PSD-95 or SYP clustering and does not appear to impact excitatory synapse density in hippocampal neurons.

### Spine morphology and dendritic complexity are unaltered in wtHTT-reduced hippocampal neurons

**Figure 3:**
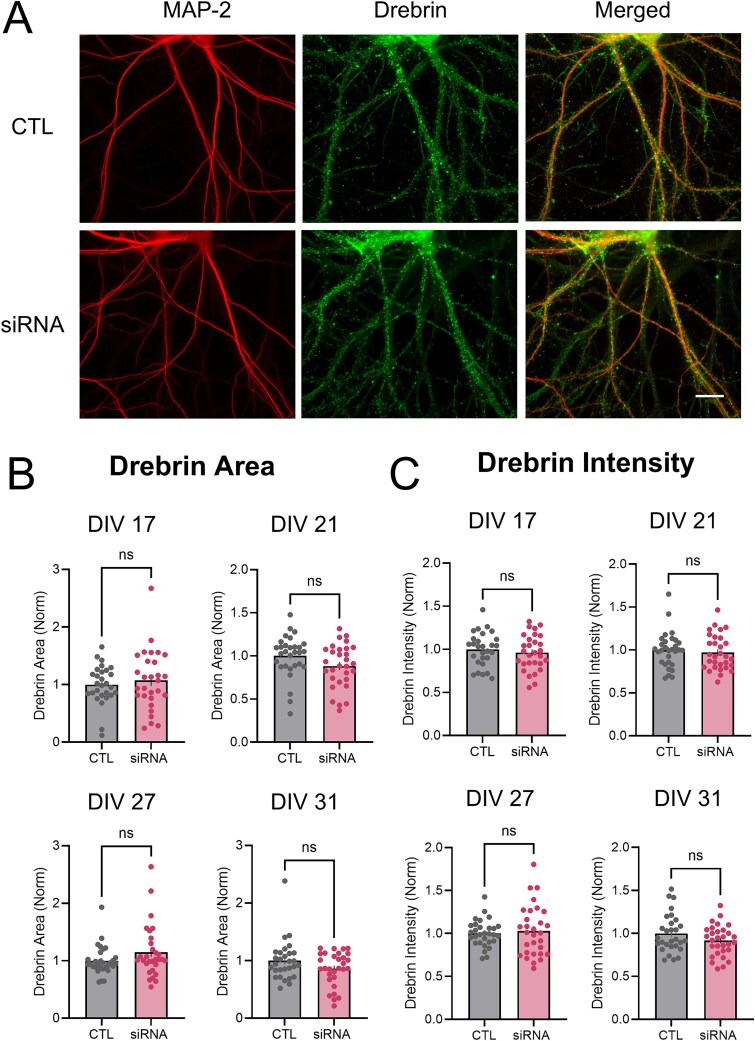
(A) dendritic processes and spines visualized by immunocytochemical staining with MAP-2 and drebrin, respectively. Scale bar represents 10 μm. (B) analysis of drebrin area values. (C) analysis of drebrin intensity values. Data were assessed by unpaired t-tests and Mann–Whitney tests and are represented as mean ± SEM. Ns: Nonsignificant.

**Figure 4:**
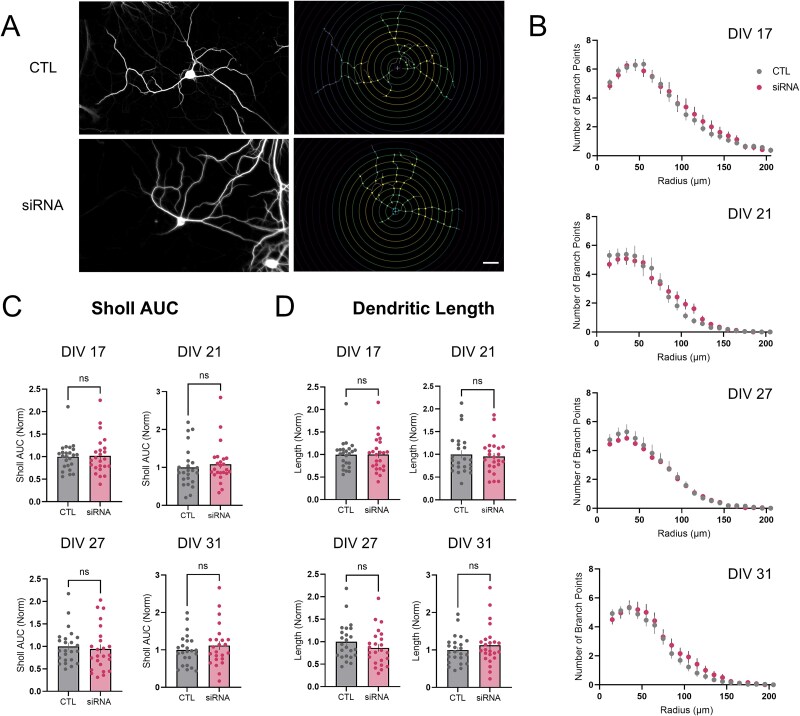
(A) left: Dendritic processes visualized by MAP-2 staining. Right: Corresponding ROI traces and branch points identified by Sholl analysis (FIJI). Scale bar represents 20 μm. (B) dendritic complexity values quantified using Sholl analysis. (C) area under the curve (AUC) values from Sholl complexity graphs. (D) analysis of total dendritic length quantified from manual ROI traces. Data were assessed by two-way RM ANOVAs and Mann–Whitney tests and are represented as mean ± SEM. Ns: Nonsignificant.

We next investigated the effect of wtHTT knockdown on spine morphology, as the morphology and density of dendritic spines were previously reported to be altered when wtHTT was deleted embryonically in the mouse cortex [25]. Here, we found that loss of wtHTT in mature hippocampal neurons did not lead to changes in dendritic spine morphology, as quantified by drebrin staining (Fig. 3A) [26]. siRNA treatment had no significant effect on the drebrin-positive area on or adjacent to MAP-2-positive neurons (Fig. 3B, DIV 17 *P* = 0.757; DIV 21 *P* = 0.053; DIV 27 *P* = 0.112; DIV 31 *P* = 0.309), nor did it have any measurable effect on the average drebrin intensity (Fig. 3C, DIV 17 *P* = 0.479; DIV 21 *P* = 0.569; DIV 27 *P* = 0.624; DIV 31 *P* = 0.113). We also assessed overall dendritic arborization by Sholl analysis in our cultured hippocampal neurons (Fig. 4A). wtHTT knockdown also had no effect on dendritic complexity (Fig. 4B, DIV 17 *P* = 0.783; DIV 21 *P* = 0.860; DIV 27 *P* = 0.693; DIV 31 *P* = 0.453), AUC of the Sholl analysis graphs (Fig. 4C DIV 17 *P* = 0.834; DIV 21 *P* = 0.376; DIV 27 *P* = 0.398; DIV 31 *P* = 0.584), or total dendritic length (Fig. 4D, DIV 17 *P* = 0.878; DIV 21 *P* = 0.992; DIV 27 *P* = 0.222; DIV 31 *P* = 0.443). Overall, these data suggest that dendritic spines and overall dendritic complexity in hippocampal neurons are not affected by the magnitude and duration of wtHTT knockdown used in the present study.

### Loss of wtHTT increases nuclear to soma size ratio of primary neurons

**Figure 5:**
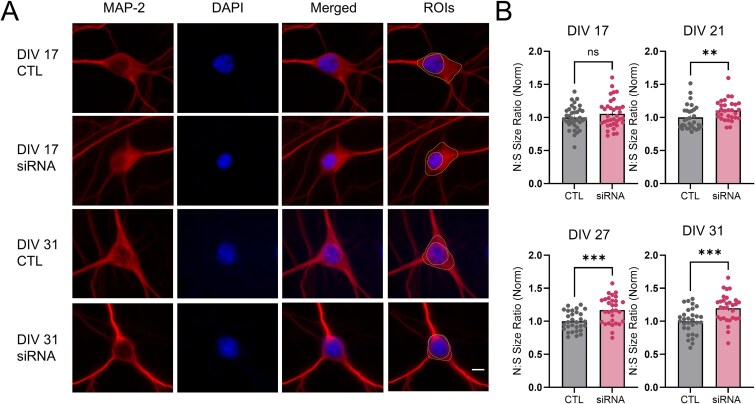
(A) representative MAP-2 and DAPI immunocytochemically stained primary hippocampal neurons. Nuclear to soma size ratios (N:S) were quantified from manual ROI traces. Scale bar represents 5 μm. (B) analysis of N:S ratios. Data were assessed by unpaired t-tests and Mann–Whitney tests and are represented as mean ± SEM. ^*^^*^P < 0.01, ^*^^*^^*^P < 0.001.

Finding relatively little effect of wtHTT reduction on synaptic and dendritic measures in hippocampal neurons, we next turned our attention to the nucleus, as transcriptional dysregulation is a molecular hallmark of HD pathogenesis [22] and wtHTT is known to interact with numerous transcription factors and epigenetic regulators [19, 27–29]. Furthermore, nuclear dysfunction and altered nuclear morphology have been recently reported in HD tissues [30, 31]. Given that we saw no obvious trends in total nuclear size of all nuclei regardless of cell type (Fig. S4A) and variable results with no obvious trends across *in vitro* timepoints for both raw nuclear size (Fig. S5A) and raw cell body size of MAP-2 positive neurons (Fig. S5B), we decided to quantify nuclear size relative to the soma size within MAP-2 labelled neurons (N:S size ratio). In response to wtHTT knockdown, we observed a gradual and highly consistent increase in nuclear size relative to the soma size in MAP-2 positive neurons as these cells matured from DIV 17 to DIV 31 (Fig. 5A and B). While DIV 17 wtHTT-lowered cells did not differ from controls in their N:S size ratio (*P* = 0.232), DIV 21 (*P* = 0.006), DIV 27 (*P* = 0.0004) and DIV 31 (*P* = 0.0004) neurons all had significantly larger nuclei relative to their cell bodies following wtHTT siRNA treatment (Fig. 5B).

### wtHTT knockdown alters heterochromatin architecture in hippocampal neurons

**Figure 6:**
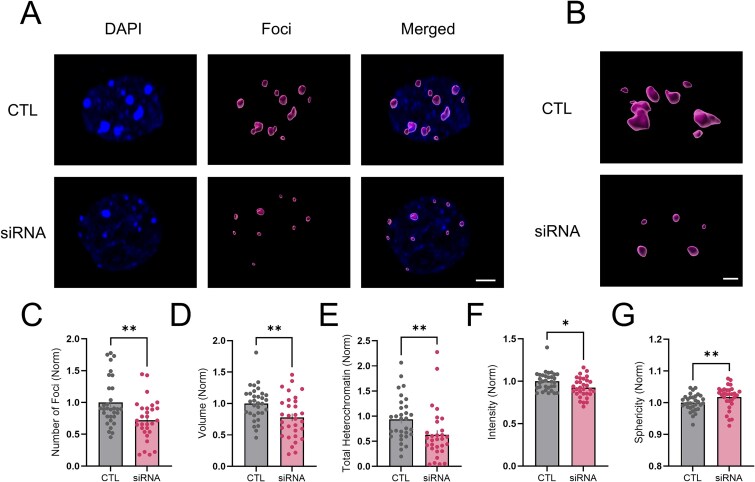
(A) representative 3D DAPI stained nuclei and heterochromatic foci imaged by Airyscan microscopy. Foci were identified using Imaris software based on thresholding DAPI hotspots to average nuclear intensity levels. Scale bar represents 3 μm. (B) representative examples of enlarged DAPI hotspots. Scale bar represents 1 μm (C) analysis of foci density. (D) analysis of foci volume. (E) analysis of total heterochromatin. (F) analysis of foci intensity. (G) analysis of foci sphericity. Data were assessed by unpaired t-tests and Mann–Whitney tests and are represented as mean ± SEM. ^*^P < 0.05, ^*^^*^P < 0.01.

Enlarged nuclei with respect to the cell body may be indicative of altered chromatin structure in hippocampal neurons following wtHTT reduction [32]. Indeed, wtHTT has been shown to interact with numerous chromatin organizers [27–29]. As wtHTT knockdown increased the N:S size ratio in our cultured hippocampal neurons, we wondered whether a sensitive super-resolution 3D imaging approach might be able to detect early dysregulation of chromatin structure. At our earliest experimental time point of DIV 17, we imaged individual nuclei from hippocampal neurons using Airyscan confocal microscopy and quantified heterochromatic foci, identified as areas of high DAPI intensity as described previously [33] (Fig. 6A and B). Here, we observed clear evidence for heterochromatin structural abnormalities following wtHTT knockdown (Fig. 6C–G). siRNA treatment decreased the number (Fig. 6C, *P* = 0.004) and the volume (Fig. 6D, *P* = 0.005) of individual heterochromatic foci, thereby decreasing the amount of total heterochromatin found in neuronal nuclei (Fig. 6E, *P* = 0.001). DAPI intensity was also significantly lower in the heterochromatic regions following wtHTT reduction (Fig. 6F, *P* = 0.013). siRNA treatment was also found to alter the shape of heterochomatic foci, as we observed a significantly increased sphericity (Fig. 6G, *P* = 0.007). These results suggest that wtHTT helps maintain heterochromatin stability and that wtHTT reduction may promote neuronal nuclei to take on a more transcriptionally active, euchromatin-like state.

### Loss of wtHTT leads to progressive overactivation of CREB within nuclei

**Figure 7:**
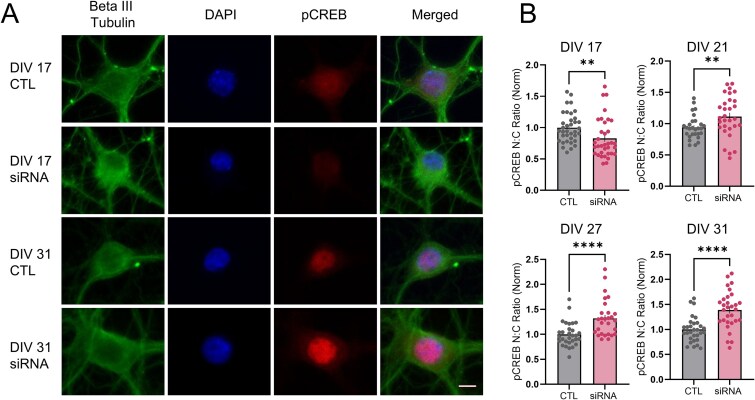
(A) representative beta III tubulin, DAPI and phosphorylated CREB (pCREB) immunostained cells used to quantify nuclear to cytosolic (N:C) pCREB intensity ratios. Scale bar represents 5 μm. (B) analysis of N:C pCREB intensity ratios. Data were assessed by unpaired t-tests and Mann–Whitney tests and are represented as mean ± SEM. ^*^^*^P < 0.01, ^*^^*^^*^^*^P < 0.0001.

Due to our novel finding that loss of wtHTT leads to a progressive enlargement of the nuclear size relative to the cell body and a more relaxed euchromatic state, we next asked whether transcriptional activity might be similarly dysregulated over time. cAMP response element (CRE)-dependent transcription was previously found to be upregulated in the hippocampus of the R6/2 mouse model of HD [34]. Therefore, we decided to assess activated CRE binding protein (CREB) levels in our cultured hippocampal cells following wtHTT knockdown. For these experiments, beta-III-tubulin was used as a neuronal marker, DAPI as a nuclear marker, and an antibody against Serine 133-phosphorylated CREB (pCREB) was to visualize the active form of CREB (Fig. 7A). Neuronal cytoplasmic and nuclear ROIs were manually traced in FIJI using the beta-III-tubulin and DAPI channels, and then superimposed on the corresponding pCREB image to generate a nuclear-to-cytosolic (N:C) ratio of pCREB intensity for individual neurons (Fig. 7A). Similar to our previous results demonstrating a progressive increase in N:S size ratio following wtHTT reduction, we found that the N:C pCREB intensity ratio also increased in our wtHTT-reduced neurons, particularly at later DIVs (Fig. 7B). Initially, DIV 17 (*P* = 0.002) neurons had decreased N:C pCREB levels; however, an opposite effect was observed at later timepoints where DIV 21 (*P* = 0.009), DIV 27 (*P* < 0.0001) and DIV 31 (*P* < 0.0001) cells had significantly increased pCREB N:C ratios. Compromised integrity of the nuclear pore complex was previously shown in HD tissues, where MAP2—typically a cytosolic protein—was shown to leak into the nucleus [31]. We did not observe such nuclear pore complex breakdown following wtHTT knockdown, as the N:C ratio of MAP2 intensity was comparable between control and siRNA treated cells (Fig. S6A and B, DIV 17 *P* = 0.100; DIV 21 *P* = 0.981; DIV 27 *P* = 0.905; DIV 31 *P* = 0.498). Together, these results suggest that wtHTT knockdown results in a progressive increase in CREB activation in the nucleus of hippocampal neurons, in the absence of major alterations to the integrity of the nuclear pore complex.

### Loss of transcriptional repression observed in wtHTT reduced cultures and wtHTT conditional knockout mice

**Figure 8:**
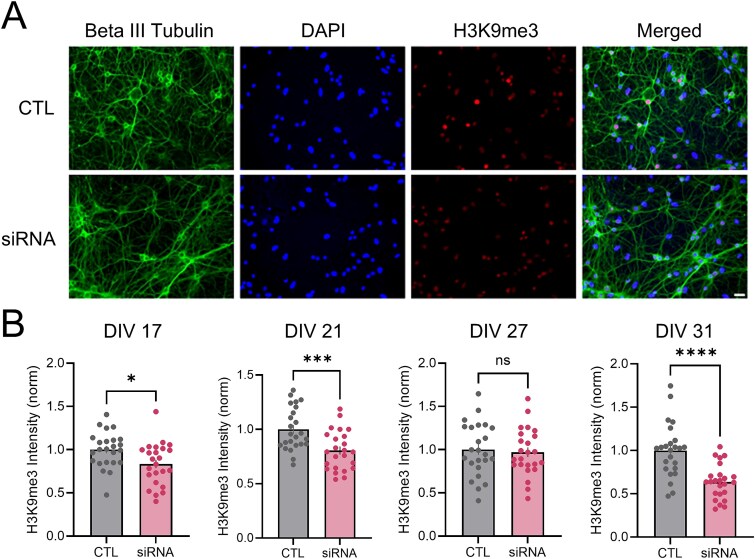
(A) representative beta III tubulin, DAPI and H3K9me3 immunostained cells. Scale bar represents 20 μm. (B) analysis of nuclear intensity levels of H3K9me3, transcriptional repression marker. Data were assessed by unpaired t-tests and are represented as mean ± SEM. ^*^P < 0.05, ^*^P < 0.001, ^*^^*^^*^^*^P < 0.0001.

**Figure 9:**
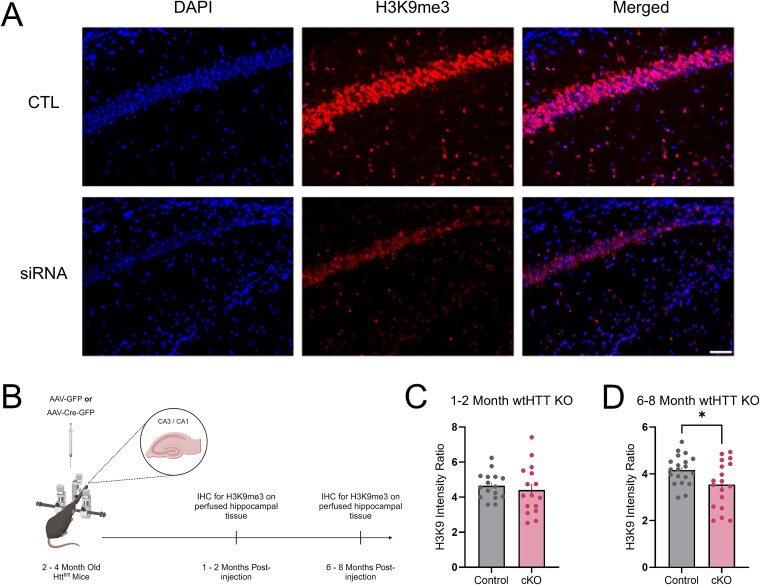
(A) representative examples of perfused slices immunostained with transcriptional repression marker, H3K9me3 and nuclear marker DAPI. (B) schematic of wtHTT conditional KO mouse model. (C) ratio of H3K9me3 nuclear ROI intensity to background in 1–2 month wtHTT KO mice. (D) ratio of H3K9me3 nuclear ROI intensity to background in 6–8 month wtHTT KO mice. ROIs generated using FIJI thresholding protocol. Data were assessed by unpaired t-tests and Mann–Whitney tests and are represented as mean ± SEM. ^*^P < 0.05.

Lastly, we wanted to assess if loss of wtHTT may be leading to a more widespread change in transcriptional activity. To investigate this question, we next measured nuclear levels of H3K9me3, a histone methyltransferase associated with constitutive heterochromatin and a common marker of transcriptional repression [35] (Fig. 8A). Interestingly, nuclear H3K9me3 levels were found to be decreased at almost all examined timepoints (DIV 17 *P* = 0.014; DIV 21 *P* = 0.001; DIV 31 *P* = < 0.0001), with the exception of DIV 27 (*P* = 0.720), supporting the idea that loss of non-pathogenic HTT can induce a more transcriptionally active state in hippocampal neurons. We also investigated whether this finding could be recapitulated in a living system using an *in vivo* mouse model. For these experiments we used wtHTT-floxed (*Htt*^fl/fl^) mice as previously described [16]. Briefly, these mice received bilateral hippocampal injections of CaMKII-promoted Cre-recombinase viral vector AAV-GFP-Cre specifically targeting dorsal CA1 at 2–4 months of age to conditionally KO (cKO) wtHTT in excitatory hippocampal neurons (Fig. 9A and B). We found that H3K9me3 levels were similar between wtHTT KO mice and controls at our earlier timepoint of 1–2 months post-Cre injection (Fig. 9C, *P* = 0.549). However, we observed a significant decrease in nuclear H3K9me3 levels in wtHTT KO animals at the later timepoint of 6–8 months post-injection (Fig. 9D, *P* = 0.024). We also quantified the expression of H3K27me3, a marker of facultative heterochromatin, at this timepoint after 6–8 wtHTT cKO (Fig. S7A). H3K9me and H3K27me together represent two of the most well-studied markers of heterochromatin in mammalian cells [36]. Similar to the results obtained for H3K9me3, we found that H3K27me3 levels were also decreased in CA1 nuclei of wtHTT cKO mice (Fig. S7B, *P* = 0.0016). These data provide *in vivo* evidence supporting our *in vitro* primary culture findings that wtHTT loss of function leads to a significant loss of transcriptional repression in hippocampal neurons.

## Discussion

Here, we examined the consequences of 1–3 weeks of wtHTT knockdown in primary hippocampal cultures, from the level of the soma and nucleus down to nanoscale properties of synaptic structure. Unexpectedly, we found that wtHTT-reduced cells showed clear nuclear dysregulation in terms of nucleus structure and transcriptional activity, while more distal morphologies, including nanoscale synaptic architecture, remained largely intact. These results suggest that the nucleus is particularly vulnerable to wtHTT-lowering, at least in mature hippocampal neurons. Furthermore, our study highlights the need to better understand the contribution of wtHTT loss of function to transcriptional dysregulation in HD, and the potential consequences of non-selective HTT lowering on gene expression profiles in various brain regions.

### Short-term wtHTT reduction appears to be well tolerated at the synaptic level in hippocampal neurons

We began our characterization of wtHTT loss on hippocampal neuronal morphology by assessing common markers of pre- and postsynaptic sites, as synaptic dysfunction is known to occur years before cell death in HD and may help predict subsequent neuronal degeneration and symptom onset [37]. We have also previously reviewed the abundance of literature highlighting the putative roles of wtHTT in synaptic function [15], and have recently reported that when wtHTT is conditionally deleted in the hippocampus of young adult mice for 1–2 months, LTP and hippocampal-dependent learning are impaired [16]. As well, previous studies have shown that wtHTT interacts with PSD-95 and that wtHTT reduction decreases PSD-95 cluster size in corticostriatal co-cultured neurons [38, 39]. Glutamate receptor clustering was also shown to be altered in Q175FDN heterozygous HD mice, both basally and in response to plasticity-inducing chemical stimulation, one of a number of demonstrations that HTT mutation alters synaptic protein organization [24]. Here, synaptic structures, specifically excitatory postsynaptic sites visualized by PSD-95 and presynaptic terminals visualized by SYP ICC, were investigated using SRRF, a super-resolution microscopy protocol compatible with conventional microscopes and fluorophores [40]. While we did not observe any changes in the nanoscale clustering or density of these synaptic proteins, the current study did not measure possible functional changes in synaptic transmission. It was recently shown that activity-dependent synaptic vesicle (SV) endocytosis is slowed in a region-dependent manner following wtHTT reduction [41]. Thus, while synaptic structure—as assessed in the present study—appeared largely normal in wtHTT-reduced neurons, evidence from prior studies suggest that at least some aspects of synaptic transmission can be compromised following wtHTT loss in primary culture.

We found that up to three weeks of wtHTT reduction by siRNA in primary hippocampal cultures did not alter gross dendrite morphology in terms of dendritic complexity and length, nor did it alter spine density as quantified by drebrin ICC. These results may seem in contrast to previous findings in the literature; however, one would expect the consequences of wtHTT loss of function to be complex, and highly dependent on the timing of knockdown initiation, the duration and magnitude of knockdown, as well as the brain region and specific cell types under study. A previous study demonstrated that when wtHTT knockdown was initiated at DIV 4—an earlier timepoint than that used in the present study—reduced dendritic complexity was observed in primary hippocampal cells just three days later [42]. Such a finding may result from a slower maturation of dendritic complexity at those early DIVs rather than a loss of previously established mature neurites. Conditional knockout of wtHTT at two months of age has previously been shown to reduce dendritic complexity when assessed six months later, although this analysis was restricted to newborn neurons expressing doublecortin [43]. Knocking out wtHTT in the cortex was shown to induce complex effects on cortical neuron structure. Increased dendritic complexity was initially observed at P21 in layer five neurons, but complexity returned to control levels by P35 [44]. On the other hand, layer 2/3 wtHTT KO cortical neurons had significantly reduced complexity at both P21 and P35 time points [44]. Thus, while dendritic complexity appeared largely normal in the present study, evidence from prior work suggest that in some cell types and certain experimental conditions, wtHTT reduction can indeed alter dendritic arborization, particularly during periods of neuronal maturation.

### wtHTT as a regulator of nuclear and chromatin structure in hippocampal neurons

Here, we show that nuclear size progressively increases relative to cell body size in wtHTT-reduced cells, which we chose to further investigate as elevated N:S size ratios are associated with disease states including cancer [45]. The nuclear to cytoplasmic size ratio, also known as the karyoplasmic ratio, is generally maintained throughout the cell cycle phases in dividing cells; however, this ratio is increased in certain cancerous cells [46, 47]. It is interesting to note that both HD and cancer cells share the molecular pathogenic hallmark of transcriptional dysregulation [48, 49]. Additionally, both expression of mHTT and low levels of wtHTT are associated with tumor metastasis in breast cancer [50, 51]. In HD animal models, nuclear morphology has been shown to be altered in R6/1 mice [30], and nuclear lamina structural proteins, particularly lamin-B1, display abnormal expression and organization in both HD mice and postmortem tissues from HD patients [30, 52]. In addition to an enlarged N:S ratio, our results also showed that loss of wtHTT led to altered chromatin compaction within neuronal nuclei. Specifically, following siRNA-mediated wtHTT knockdown we found fewer heterochromatic foci that were smaller and more spherical with lower DAPI intensity levels, suggesting a loss of DNA compaction in these neurons. wtHTT has been previously implicated in chromatin remodelling through its interaction with the epigenetic modifier polycomb repressive complex 2 (PCR2) [29]. As well, wtHTT directly interacts with the transcriptional regulator MeCP2, and both loss and overexpression of the MeCP2 protein significantly alters the 3D structure of chromatin within mouse neuronal nuclei [28, 33, 53]. wtHTT has also been shown to bind directly to CBP, with this association being much stronger and dysfunctional when bound to mHTT [20, 27]. CBP is a histone acetyltransferase (HAT) that can enhance chromatin availability for gene transcription. Additionally, wtHTT interacts with numerous other transcriptional regulators including nuclear factor kappa B (NF-κB), tumor protein p53 and repressor-element 1-silencing transcription/neuron-restrictive silencer factor (REST/NRSF), to name just a few (reviewed in [21]). Very recently, wtHTT loss of function has also been reported to contribute to transcriptional dysregulation in HD based on RNA-sequencing data from human iPSC models [54].

We observed a progressive decrease in H3K9me3 levels in both wtHTT-reduced primary neuronal cultures and a wtHTT-depleted conditional knockout mouse model. In contrast, elevated H3K9me3 expression has been observed in a striatal HD cell line [55], in the striatum of R6/2 mice [56], and in the cortex and caudate of postmortem tissue from HD patients [57]. In embryonic stem cells, wtHTT knockdown enhanced H3K9me3 levels, and this effect persisted when these cells were differentiated into neural progenitor cells [58]. Mechanistically, in human embryonic stem cells it was shown that wtHTT normally binds the chromatin regulator ATF7IP, inhibiting an ATF7IP complex with the H3 histone methyltransferase SETDB1 and thereby maintaining low H3K9me3 levels [58]. A limitation of the present study is that we did not investigate the mechanisms by which wtHTT reduction can decrease H3K9me3 and heterochromatin in mature hippocampal neurons, although it appears likely that HTT lowering may affect histone methylation and chromatin architecture in a cell type- and/or brain region-dependent manner. Further studies are required to clarify whether wtHTT can directly influence chromatin structure, or if the observed effects are indirect, resulting from disruptions in the broader cellular functions mediated by wtHTT [21]. A recent study mapped the HTT genomic occupancy and discovered thousands of reproducible sites of HTT-chromatin interactions in the mouse which were altered by HTT mutation. Their data suggest that altered wtHTT-chromatin interactions may underlie the transcriptional dysregulation in HD, and that transcriptional dysregulation in HD is likely driven by a combination of mHTT gain of function and wtHTT loss of function [59]. Interestingly, alterations in chromatin architecture have been highlighted in recent years as an early molecular change correlating with aging that may contribute to many aging-related nuclear deficits [60, 61]. Thus, it is possible that wtHTT loss may accelerate the aging process within these cells, leading to the gradual increase in N:S size ratio seen in our wtHTT-lowered neurons.

H3K9me3 is traditionally recognized as a marker of constitutive heterochromatin, but it also plays an increasingly recognized role in regulating cell-type identity by silencing lineage-inappropriate genes [62]. In the present study, we also observed a significant reduction of H3K27me3 following wtHTT depletion in the adult hippocampus. As H3K27me3 is a hallmark indicator of facultative heterochromatin, these results raise the possibility that hippocampal neurons may begin to lose their identity following wtHTT loss by expressing developmentally inappropriate and/or cell type inappropriate genes. Investigating the genome-wide consequences of wtHTT reduction on RNA expression in hippocampal neurons is therefore of great interest for future studies.

### wtHTT loss bidirectionally impacts nuclear pCREB

Our findings also reveal a dynamic and biphasic impact of wtHTT reduction on CREB activity. We observed an initial decrease of nuclear pCREB at DIV17, which later transitioned to elevated levels with sustained wtHTT knockdown. The early reduction in pCREB may be explained by diminished BDNF–TrkB signaling, consistent with wtHTT’s established roles in BDNF production and trafficking [11, 13, 63]. Over time, the progressive chromatin decompaction we observe could allow a more permissive environment for CREB binding to CRE DNA sequences, which can stabilize CREB’s phosphorylated form by protecting it from PP1-mediated dephosphorylation [64]. Furthermore, given wtHTT’s interaction with CBP [27], wtHTT loss could increase CBP availability which would further boost CREB activity. Considering wtHTT’s extensive interactome [6], numerous other mechanistic possibilities could contribute to the observed effects on pCREB, including alterations in synaptic neurotransmission and signaling downstream of NMDAR activation [65]. Future studies will focus on elucidating the mechanisms underlying the observed effects of wtHTT reduction on CREB signaling and nuclear/chromatin structure. It is also of great interest to determine how much wtHTT lowering can be tolerated in the adult hippocampus and elsewhere, before giving rise to the morphological and transcriptional deficits reported here.

## Conclusions

The role of endogenous wtHTT in maintaining neuronal function in adulthood has become more appreciated in recent years; however, the consequences of its loss in the adult brain and particularly outside the corticostriatal pathway remain understudied. Uncovering how wtHTT functions in the adult brain is imperative given the emergence of non-selective HTT therapeutics in clinical trials. Here, we demonstrate that loss of wtHTT alters nuclear structure and significantly impacts chromatin architecture where neuronal nuclei contained fewer and smaller heterochromatic foci. In line with these findings, we also observed that wtHTT reduction led to altered transcriptional activity in both *in vitro* and *in vivo* models. The precise mechanisms underlying the presently observed effects, and the downstream consequences of the observed nuclear and chromatin changes are of great interest for future research. Overall, our findings highlight the nucleus as an organelle that appears to be particularly sensitive to HTT lowering and suggests that HTT reduction may itself drive transcriptional dysregulation.

## Materials and methods

### Animals

For primary culture experiments, timed-pregnant Sprague Dawley rats were purchased from Charles River Laboratory (Saint-Constant, QC, Canada). Conditional knockout (KO) mice used for experiments were initially provided by Dr Scott Zeitlin, University of Virginia, Charlottsville, VA. Breeding colonies were then established and maintained at Memorial University’s Animal Care facility. Mice were biologically engineered to have their endogenous *wtHtt* alleles flanked with loxP sites (*Htt*^fl/fl^) in order to conditionally inactivate the mouse *Htt* gene in a homozygous manner by Cre expression. Mice were bred as homozygous x homozygous. Both male and female animals were used for all experiments. Animals were housed in ventilated cage racks and kept on a 12 h light/dark cycle (lights on at 7:00 a.m.) with food and water available ad libitum. All experimental procedures were approved by Memorial University’s Institutional Animal Care Committee and were performed in accordance with the guidelines set by the Canadian Council on Animal Care.

### Materials

Self-deliverable, cholesterol conjugated siRNA against HTT was custom ordered from Advirna (Worcester, MA, USA) and designed as previously described [66] (Table 1). siRNAs used here have been previously shown to have robust uptake and efficacy in primary neurons [67]. A scrambled, non-target control siRNA was used for comparison throughout and is herein referred to as ‘CTL’. Pilot experiments demonstrated that simply adding the self-deliverable siRNA to the culture media, regardless of whether it was targeted against wtHTT or a scrambled control, enhanced the dendritic arborization of primary cultured neurons (Fig. S1). For this reason, we did not use a media only control group and rather chose to compare the effects of the wtHTT siRNA to that of the scrambled siRNA, with the latter serving as our CTL group throughout. The following primary antibodies were used for immunocytochemistry (ICC): glial fibrillary acidic protein (GFAP, 1:500, MA5–12023), ionized calcium-binding adapter molecule 1 (IBA1, 1:500, 019–19 741), microtubule-associated protein 2 (MAP2, 1:2000, AB5622), βIII-tubulin (1:500, T8660), phosphorylated cAMP response element-binding protein (pCREB, 1:250, 9198S), drebrin (1:500, ab60932), postsynaptic density protein 95 (PSD-95, 1:500, MA1045), synaptophysin (SYP, 1:1000, ab32594). The following primary antibodies were used for immunohistochemistry (IHC): trimethylated histone H3 (Lys9) (H3K9me3, 1:1000, ab8898), trimethylated histone H3 (Lys27) (H3K27me3, 1:500, 07–449). The following secondary antibodies were used: Alexa Fluor 488 (1:500, A-11029), and Alexa Fluor 594 (1:500, A-11037).

**Table 1:** Modification of self-deliverable siRNA.

*siRNA ID*	*Unformatted Sequence (5′-3′)*	*Formatted Sequence*
HTT10150_AS	UUAAUCUCUUUACUGAUAUA	[5Phos][mU][pS][fU][pS][mA][fA][mU][fC][mU] [fC][mU][fU][mU][fA][mC][fU][pS][mG][pS][fA] [pS][mU][pS][fA][pS][mU][pS][fA]
HTT10150_S	CAGUAAAGAGAUUAA	[fC][pS][mA][pS][fG][mU][fA][mA][fA][mG][fA] [mG][fA][mU][fU][pS][mA][pS][fA][3'CholTEG]
NTC3_AS	UAGGAAAACAUGUAAACCAA	[5Phos][mU][pS][fA][pS][mG][fG][mA][fA][mA] [fA][mC][fA][mU][fG][mU][fA][pS][mA][pS][fA] [pS][mC][pS][fC][pS][mA][pS][fA]
NTC_S	UUACAUGUUUUCCUA	[fU][Ps][mU][Ps][fA][mC][fA][mU][fG][mU][fU] [mU][fU][mC][fC][Ps][mU][Ps][fA][3'CholTEG]

Chemical modifications are defined as follows: “f”—2′ Fluoro, “m”—2′ O-methyl, “Ps”—phosphorothioate linker, “5Phos”—5′ phosphate, “3’CholTEG”—Cholesterol conjugate

### Primary neuronal culture

Cultures of primary hippocampal neurons were prepared from postnatal day 1 (P1) male and female rat pups as previously described [68]. Briefly, hippocampi were dissected. The tissues were digested with papain (Millipore-Sigma, Canada) and DNase at 37°C for 30 minutes. The digested tissues were inactivated by the addition of ovomucoid (Worthington Biochemical Corp, Lakewood, USA) at 37°C for 10 minutes. Tissue fragments were subjected to mechanical dissociation by repeated trituration using a fire-polished glass pipette. The suspension was pelleted and the dissociated hippocampal cells were seeded in plating medium at 60000 cells to 12-mm coverslips in a 24 well plate or 1 million cells per well of a 6 well plate. The medium was replaced with serum-free medium (Neurobasal plus medium with 1% B27 plus, 1% Penicillin–Streptomycin, 0.25% Glutamax) within 2–6 h. Ara-C was added to cell culture after 2 days *in vitro* (DIV 2) at a final concentration of 5 μM. At DIV 10 (^+/−^one day) cells were treated with 0.4 μM self-deliverable siRNA (Advirna) against HTT. These siRNAs were cholesterol conjugated to achieve unassisted cellular uptake and were previously validated [66]. DIV 17, 21, 27 and 31 (^+/−^ one day for each time point) primary hippocampal cells were fixed in a 4% paraformaldehyde (PFA) with 4% sucrose in PBS for 15 minutes followed by 3–4 rounds of 10-minute PBS washes.

### Western blotting

Hippocampal neurons were collected and pelleted (500 ×g, 5 minutes, 4°C). Proteins were extracted with lysis buffer (50 mM Tris pH 8.0, 150 mM NaCl, 1% NP40, 10% glycerol, 1 mM sodium orthovanadate, 1 mM NaF, 2 mM MgCl_2_, 25 mM O-β-thioglucopyranoside) and 1X Roche complete protease inhibitor. Protein samples were cleared of debris by centrifugation (20 000 xg, 10 minutes, 4 degrees Celsius). The protein concentration was determined by the Bradford method (Bio-Rad, Missisauga, Ontario, Canada). Samples were prepared by denaturing the lysate in Bolt LDS sample buffer (4×) with 100 mM DTT and heated at 70°C for 10 minutes. The samples were resolved on 4–12%, Bis-Tris, Bolt gels (Invitrogen #NW04120BOX) and transferred to 0.45 μm nitrocellulose membrane overnight at 24 volts for 16 h with transfer buffer containing 10% methanol. The membrane was blocked with 5% milk in TBS, and then incubated with anti-HTT antibody (Millipore cat#MAB 2166, 1:1000 dilution) and anti-beta actin (Sigma cat#A5316) overnight at 4°C. The membrane was washed and incubated with peroxide-conjugated goat anti-mouse (Pierce #31430, 1:5000 dilution). The bands were detected by SuperSignal West Pico Plus chemiluminescent substrate (Thermo Scientific #34580).

### Immunocytochemistry

The ICC protocol consisted of incubating cells for 1 h on a shaker at room temperature in a blocking solution consisting of 5% bovine serum albumin (BSA) and 0.2% Triton-X in 1xPBS. After blocking, primary antibodies were diluted in a 0.2% Triton-X 1xPBS solution and incubated overnight on a shaker at 4°C. On the second day, cells received 3–4 rounds of 10-minute 1xPBS washes and secondary antibodies were then diluted in a 0.2% Triton-X 1xPBS solution and incubated in the dark for 1.5 h on a shaker at room temperature. Cells then received 3–4 rounds of 10-minute 1xPBS washes and were mounted using Fluoroshield mounting medium with DAPI to visualize nuclei.

### Sholl analysis

Dendrites were manually traced in FIJI (ImageJ) based on MAP2 staining and saved as individual regions of interest (ROIs). ROIs for each cell were then copied to a new image and Sholl analysis was performed on these tracings using the Neuroanatomy Sholl plugin available for FIJI. The following Sholl analysis settings were used: start radius: 5 μm; step size: 10 μm; end radius: 205 μm. Dendritic complexity was recorded as the number of dendritic branch points identified by Sholl analysis. Sholl area under the curve (AUC) values were calculated in GraphPad Prism. Total dendritic length was also quantified for each neuron based on the total length of the MAP2 dendrite ROI traces.

### Super-resolution radial fluctuation and Airyscan confocal imaging

Fixed hippocampal neurons stained for PSD-95 and SYP were imaged using an Olympus BX51 microscope (60x oil immersion objective, 1.4 NA) equipped with an EM-CCD camera (Andor, iXon Ultra 897) and analyzed following a similar protocol as described previously by our group [24]. Briefly, 100 consecutive images were taken of PSD-95 and SYP-stained dendritic segments using Andor Solis software and super-resolution was achieved by incorporating the NanoJ-Super-Resolution Radial Fluctuations (SRRF) plugin available on FIJI [69]. SRRF images of PSD-95 and SYP were converted to binary images using local thresholding in FIJI as previously described [24]. The analyze particles function in FIJI was then used to calculate average puncta size as well as puncta density within manually traced dendrite ROIs. Puncta ROIs were then superimposed on the non-binary SRRF image to quantify average puncta intensity. 3 dimensional (3D) morphology of the DAPI stained neuronal nuclei were imaged using Zeiss Airyscan confocal microscopy to visualize heterochromatic foci [33]. 0.14 μm interval Z-stack images were taken of individual nuclei (63x oil immersion objective, 1.4 NA) and heterochromatic foci within each nucleus were then analyzed.

### Imaris image analysis

Imaris software (version 9.9.0, Oxford Instruments) was used to generate ROIs of heterochromatic foci. The “Surfaces” detection method was applied using a constant threshold value and incorporated the following settings: number of voxels: above 100, diameter of largest sphere: 0.3 μm. Imaris ‘Surfaces” detection analysis was also used to identify drebrin stained segments within close proximity to MAP2 neuronal signal. An initial “Surfaces” protocol was applied to the MAP2 channel using a set intensity threshold value to generate ROIs of MAP2 stained structures. A second “Surfaces” protocol was then applied to the drebrin channel using a set intensity threshold value to generate ROIs of drebrin stained structures, and a filter was then applied to these drebrin ROIs with the setting: “Shortest distance to surfaces”: below 3 μm away from the MAP2 surfaces ROI. To account for differences in the number of dendrites in a given image, drebrin area was calculated by dividing the total drebrin-positive area by the total MAP2-positive area for each image. ROIs of whole DAPI positive nuclei were also generated in Imaris to quantify average nuclear area and cell densities. The “Surfaces” detection method was applied with manual thresholding using the following settings: “Region growing estimated diameter”: 5.00 μm; “Region growing morphological split”: true. The following filter settings were also used: “Area”: above 175 μm^2^; “Distance to image boarder”: above 1.00 μm, “Intensity mean”: above 35 AU.

### Stereotaxic surgery

*Htt*  ^fl/fl^ mice aged 2–4 months were anesthetized using isoflurane (3% induction, 1.5–2% maintenance) and injected subcutaneously in the abdominal region with 2 mg/kg meloxicam and below the scalp with 0.1 ml/0.2% lidocaine. Above the desired brain coordinates two small holes were drilled in the skull using an Ideal Micro-Drill (Harvard Apparatus). A Model 7002 KH Neuros Hamilton Syringe was used along with an infusion pump (Pump 11 Elite Nanomite, Harvard Apparatus) to inject 1 μl pENN.AAV.CamKII.HI.GFP-Cre.WPRE.SV40 (Addgene, catalog # 105551-AAV9) or 1 μl pENN.AAV.CamKII0.4.eGFP.WPRE.rBG (Addgene, catalog #105541-AAV9) bilaterally into the dorsal hippocampus (injection rate, 2 nl/s). The following coordinates were used with respect to distance from bregma: 2.6 mm posterior, 2.3 mm medial/lateral and 1.1–1.3 mm ventral to brain surface. The syringe was left in place for at least 5 min following the injection before careful removal from the brain. 0.5 ml of 0.9% saline was injected subcutaneously in the abdominal region and the scalp incision was sutured. Mice were monitored for 20–30 minutes as they recovered post-surgery on a heating pad before being returned to their designated home cage area.

### Immunohistochemistry

Mice were fixed 1–2 or 6–8 months post-Cre injection by intracardiac perfusion of 4% paraformaldehyde and whole brains were dissected. Brains were submerged in 4% PFA overnight at 4°C and were then cryoprotected in 30% sucrose. Whole brains were rapidly frozen by cold isopentane (2-methylbutane) and sliced at 20 μm by cryostat (Leica CM3050 S). Sections were mounted directly onto gelatin-coated glass slides and stored at −80°C until use. The immunohistochemistry (IHC) protocol consisted of creating a hydrophobic barrier around hippocampal slides using a DAKO Pen (Agilent Technologies, catalog # s200230–2) then washing sectioned slices in 1xPBS solution. Next, slides were incubated for 1 h at room temperature in a blocking solution consisting of 5% bovine serum albumin (BSA) and 0.2% Triton-X in 1xPBS. After blocking, primary antibodies were diluted in a 1% BSA/0.2% Triton-X PBS solution and incubated overnight on at 4°C. On the second day, slides were washed with 1xPBS and secondary antibodies were diluted in a 1% BSA/0.2% Triton-X PBS solution and incubated in the dark for 2 h at room temperature. Slides received a final PBS wash step and were coverslipped with Fluoroshield mounting medium with DAPI to visualize nuclei (Abcam, catalog # ab104139).

### Statistical analyses

Where stated, data were normalized within each culture to the average control value to account for inter-culture variability. All statistical tests were performed using version 10.2.2 GraphPad Prism. N values, specific statistical tests used, p values, F values and degrees of freedom are described in Table 2. Outliers were identified using the ROUT method (Q = 1%). P values less than 0.05 were considered significant.

**Table 2:** Statistical tests used and accompanying values.

*Figure #*	*Experiment*	*Culture/Animal (N) and sample (n)*	*Statistics performed*	*p value*	*Degrees of freedom/F (DFn, DFd)*
1B	DIV 17 wtHTT western blot	N = 5	Unpaired parametric t test	CTL vs siRNA: *P* = 0.0118	107.1 (5, 5)
1C	Culture composition experiment	N = 3, image n = 8	Unpaired parametric t test	Non-Ara-C vs Ara-C culture: *P* = < 0.0001	6.513 (7, 7)
2B	SRRF SYP density	N = 3, dendrite n = 16–18	**DIV 17:** Unpaired parametric t test **DIV 21:** Unpaired parametric t test **DIV 27:** Unpaired parametric t test **DIV 31:** Unpaired parametric t test	CTL vs siRNA: *P* = 0.3799 CTL vs siRNA: *P* = 0.7426 CTL vs siRNA: *P* = 0.5543 CTL vs siRNA: *P* = 0.2218	1.304 (17, 17) 1.171 (17, 16) 2.071 (16, 17) 1.486 (15, 17)
2C	SRRF PSD-95 size	N = 3, dendrite n = 17–18	**DIV 17:** Unpaired parametric t test **DIV 21:** Mann–Whitney test **DIV 27:** Mann–Whitney test **DIV 31:** Mann–Whitney test	CTL vs siRNA: *P* = 0.6944 CTL vs siRNA: *P* = 0.5418 CTL vs siRNA: *P* = 0.0717 CTL vs siRNA: *P* = 0.1626	3.374 (17, 17) Not reported Not reported Not reported
2D	SRRF PSD-95 intensity	N = 3, dendrite n = 17–18	**DIV 17:** Unpaired parametric t test **DIV 21:** Unpaired parametric t test **DIV 27:** Mann–Whitney test **DIV 31:** Mann–Whitney test	CTL vs siRNA: *P* = 0.2097 CTL vs siRNA: *P* = 0.8030 CTL vs siRNA: *P* = 0.1259 CTL vs siRNA: *P* = 0.0790	4.330 (17, 17) 1.861 (17, 17) Not reported Not reported
2E	SRRF PSD-95 density	N = 3, dendrite n = 17–18	**DIV 17:** Unpaired parametric t test **DIV 21:** Unpaired parametric t test **DIV 27:** Unpaired parametric t test **DIV 31:** Unpaired parametric t test	CTL vs siRNA: *P* = 0.0744 CTL vs siRNA: *P* = 0.4262 CTL vs siRNA: *P* = 0.2932 CTL vs siRNA: *P* = 0.4023	2.294 (17, 17) 2.264 (17, 17) 1.005 (17, 16) 1.040 (16, 17)
3B	Drebrin area	N = 3, dendrite n = 28–30	**DIV 17:** Mann–Whitney test **DIV 21:** Mann–Whitney test **DIV 27:** Mann–Whitney test **DIV 31:** Mann–Whitney test	CTL vs siRNA: *P* = 0.7574 CTL vs siRNA: *P* = 0.0532 CTL vs siRNA: *P* = 0.1124 CTL vs siRNA: *P* = 0.3086	Not reported Not reported Not reported Not reported
3C	Drebrin intensity	N = 3, dendrite n = 28–30	**DIV 17:** Unpaired parametric t test **DIV 21:** Mann–Whitney test **DIV 27:** Unpaired parametric t test **DIV 31:** Unpaired parametric t test	CTL vs siRNA: *P* = 0.4793 CTL vs siRNA: *P* = 0.5692 CTL vs siRNA: *P* = 0.6236 CTL vs siRNA: *P* = 0.1134	1.109 (28, 27) Not reported 3.683 (29, 29) 1.547 (28, 29)
4B	Sholl analysis	N = 3, neuron n = 24	**DIV 17:** Two-way RM ANOVA **DIV 21:** Two-way RM ANOVA **DIV 27:** Two-way RM ANOVA **DIV 31:** Two-way RM ANOVA	CTL vs siRNA: *P* = 0.7829 CTL vs siRNA: *P* = 0.8599 CTL vs siRNA: *P* = 0.6929 CTL vs siRNA: *P* = 0.4530	0.07676 (1, 48) 0.03147 (1, 50) 0.1577 (1, 52) 0.5721 (1, 50)
4C	Sholl area under the curve (AUC)	N = 3, neuron n = 24	**DIV 17:** Mann–Whitney test **DIV 21:** Mann–Whitney test **DIV 27:** Mann–Whitney test **DIV 31:** Mann–Whitney test	CTL vs siRNA: *P* = 0.8341 CTL vs siRNA: *P* = 0.3758 CTL vs siRNA: *P* = 0.3984 CTL vs siRNA: *P* = 0.5844	Not reported Not reported Not reported Not reported
4D	Dendrite length	N = 3, neuron n = 24	**DIV 17:** Mann–Whitney test **DIV 21:** Mann–Whitney test **DIV 27:** Mann–Whitney test **DIV 31:** Mann–Whitney test	CTL vs siRNA: *P* = 0.8782 CTL vs siRNA: *P* = 0.9919 CTL vs siRNA: *P* = 0.2221 CTL vs siRNA: *P* = 0.4429	Not reported Not reported Not reported Not reported
5B	Nuclear to soma size ratio	N = 3, neuron n = 30–36	**DIV 17:** Unpaired parametric t test **DIV 21:** Mann–Whitney test **DIV 27:** Unpaired parametric t test **DIV 31:** Unpaired parametric t test	CTL vs siRNA: *P* = 0.2323 CTL vs siRNA: *P* = 0.0061 CTL vs siRNA: *P* = 0.0004 CTL vs siRNA: *P* = 0.0004	1.524 (35, 35) Not reported 1.898 (29, 29) 1.174 (29, 29)
6C	Heterochromatic foci density	N = 3, nuclei n = 31–32	Mann–Whitney test	CTL vs siRNA: *P* = 0.0042	Not reported
6D	Heterochromatic foci volume	N = 3, nuclei n = 31–32	Unpaired parametric t test	CTL vs siRNA: *P* = 0.0050	1.585 (30, 31)
6E	Total heterochromatin	N = 3, nuclei n = 31–32	Mann–Whitney test	CTL vs siRNA: *P* = 0.0011	Not reported
6F	Heterochromatic foci intensity	N = 3, nuclei n = 31–32	Mann–Whitney test	CTL vs siRNA: *P* = 0.0129	Not reported
6G	Heterochromatic sphericity	N = 3, nuclei n = 31–32	Mann–Whitney test	CTL vs siRNA: *P* = 0.0073	Not reported
7B	Nuclear to cytosolic pCREB intensity ratio	N = 3, neuron n = 30–36	**DIV 17:** Outliers excluded, Mann–Whitney test **DIV 21:** Outliers excluded, Mann–Whitney test **DIV 27:** Outliers excluded, Mann–Whitney test **DIV 31:** Unpaired parametric t test	CTL vs siRNA: *P* = 0.0017 CTL vs siRNA: *P* = 0.0089 CTL vs siRNA: *P* = < 0.0001 CTL vs siRNA: *P* = < 0.0001	Not reported Not reported Not reported 2.136 (29, 29)
8B	H3K9me3 nuclear intensity	N = 3, neuron n = 24	**DIV 17:** Unpaired parametric t test **DIV 21:** Unpaired parametric t test **DIV 27:** Unpaired parametric t test **DIV 31:** Unpaired parametric t test	CTL vs siRNA: *P* = 0.0137 CTL vs siRNA: *P* = 0.0008 CTL vs siRNA: *P* = 0.7202 CTL vs siRNA: *P* = < 0.0001	1.420 (23, 23) 1.173 (23, 23) 1.279 (23, 23) 2.478 (23, 23)
9C	H3K9me3 nuclear intensity in 1–2 month wtHTT KO mice	N = 3–4, image n = 16	Unpaired parametric t test	CTL vs KO: *P* = 0.5485	3.338 (15, 15)
9D	H3K9me3 nuclear Intensity in 6–8 month wtHTT KO mice	N = 3–4, image n = 19–21	Unpaired parametric t test	CTL vs KO: *P* = 0.0241	2.511 (18, 20)
S1A	Sholl analysis Sholl AUC Total dendritic length	N = 3, neuron n = 24 N = 3, neuron n = 24 N = 3, neuron n = 24	Two-way RM ANOVA One-way ANOVA with Tukey's multiple comparisons test One-way ANOVA with Tukey's multiple comparisons test	Two-way RM ANOVA: *P* = 0.0039 One-way ANOVA: *P* = 0.0296 CTL vs NTC: *P* = 0.0694 CTL vs siRNA: *P* = 0.0424 NTC vs siRNA: *P* = 0.9756 One-way ANOVA: *P* = 0.0023 CTL vs NTC: *P* = 0.0075 CTL vs siRNA: *P* = 0.0054 NTC vs siRNA: *P* = 0.9934	6.018 (2, 71) 3.702 (2, 71) 3.404 (2, 71)
S2B	DIV 31 wtHTT western blot	N = 3	Unpaired parametric t test	CTL vs KO: *P* = 0.0127	18.92 (2, 2)
S4B	Total nuclear size	N = 3, image n = 14–18	**DIV 17:** Mann–Whitney test **DIV 21:** Unpaired parametric t test **DIV 27:** Mann–Whitney test **DIV 31:** Unpaired parametric t test	CTL vs siRNA: *P* = 0.2584 CTL vs siRNA: *P* = 0.0035 CTL vs siRNA: *P* = 0.2811 CTL vs siRNA: *P* = 0.8436	Not reported 1.022 (17, 17) Not reported 7.195 (13, 17)
S4C	Total nuclear density	N = 3, image n = 14–18	**DIV 17:** Mann–Whitney test **DIV 21:** Mann–Whitney test **DIV 27:** Mann–Whitney test **DIV 31:** Unpaired parametric t test	CTL vs siRNA: *P* = 0.9286 CTL vs siRNA: *P* = 0.8451 CTL vs siRNA: *P* = 0.2270 CTL vs siRNA: *P* = 0.6290	Not reported Not reported Not reported 2.651 (13, 17)
S5A	Nuclear size in neurons	N = 3, neuron n = 36	**DIV 17:** Outliers excluded, Mann–Whitney test **DIV 21:** Mann–Whitney test **DIV 27:** Mann–Whitney test **DIV 31:** Mann–Whitney test	CTL vs siRNA: *P* = 0.5772 CTL vs siRNA: *P* = 0.0088 CTL vs siRNA: *P* = < 0.0001 CTL vs siRNA: *P* = 0.6650	Not reported Not reported Not reported Not reported
S5B	Cell body size in neurons	N = 3, neuron n = 36	**DIV 17:** Mann–Whitney test **DIV 21:** Mann–Whitney test **DIV 27:** Mann–Whitney test **DIV 31:** Mann–Whitney test	CTL vs siRNA: *P* = 0.2790 CTL vs siRNA: *P* = 0.0008 CTL vs siRNA: *P* = 0.0426 CTL vs siRNA: *P* = 0.0234	Not reported Not reported Not reported Not reported
S6B	Nuclear to cytosolic MAP2 intensity ratio	N = 3, image n = 24–30	**DIV 17:** Unpaired parametric t test **DIV 21:** Unpaired parametric t test **DIV 27:** Outliers excluded, Mann–Whitney test **DIV 31:** Outliers excluded, Mann–Whitney test	CTL vs siRNA: *P* = 0.0997 CTL vs siRNA: *P* = 0.9806 CTL vs siRNA: *P* = 0.9048 CTL vs siRNA: *P* = 0.4979	2.038 (23, 23) 1.039 (29, 29) Not reported Not reported
S7B	H3K27me3 nuclear intensity in 6–8 month wtHTT KO mice	N = 4, image n = 16	Unpaired parametric t test	CTL vs KO: *P* = 0.0016	1.876 (7, 7)

## Supplementary Material

Supplementary_Figures_ddaf126

## CRediT statement

J.C.B. and M.P. designed the experiments; J.C.B, S.T.C., A.C.F., S.J.C, M.C.A.T., E.P.H. and F.N. conducted the experiments; J.C.B., S.T.C., A.C.F., S.J.C. and M.C.A.T. analyzed the data; J.C.B. wrote the paper with feedback from M.P.
