# Supplementary material for: Huntingtin reduction results in altered nuclear structure and heterochromatic instability
Source: Hum Mol Genet. 2025 Aug 1;34(19):1648–64. doi: 10.1093/hmg/ddaf126 (PMC12449191; doi:10.1093/hmg/ddaf126)
Supplement: Supplementary_Figures_ddaf126 [file supplementary_figures_ddaf126.pdf]

A

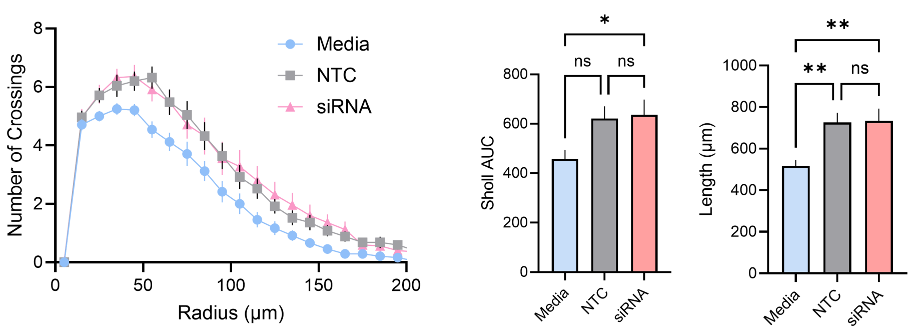

Supplementary Figure 1. A Sholl analysis, average area under the curve (AUC) values from Sholl analysis and average values for total dendritic length for DIV 17 primary neurons. Data were assessed by two-way RM ANOVAs and one-way ANOVAs with Tukey's multiple comparisons tests and are represented as mean ± SEM. ns: nonsignificant, * $p < 0.05$, ** $p < 0.01$.

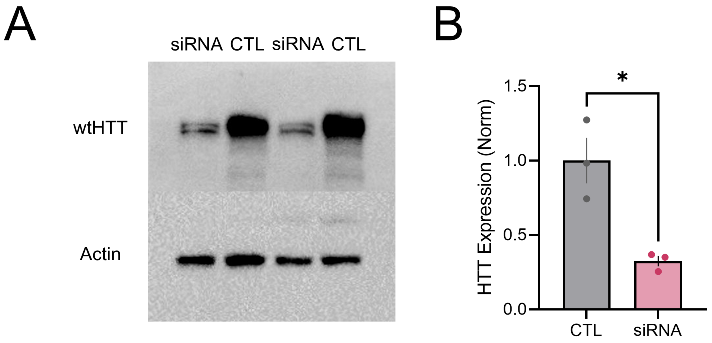

Supplementary Figure 2. **A** Left: Protein from DIV 31 hippocampal neurons was collected and wtHTT levels were assessed using Western blot. Right: Normalized wtHTT protein levels relative to actin control values. Data were assessed by unpaired t-tests and are represented as mean ± SEM. * $p < 0.05$.

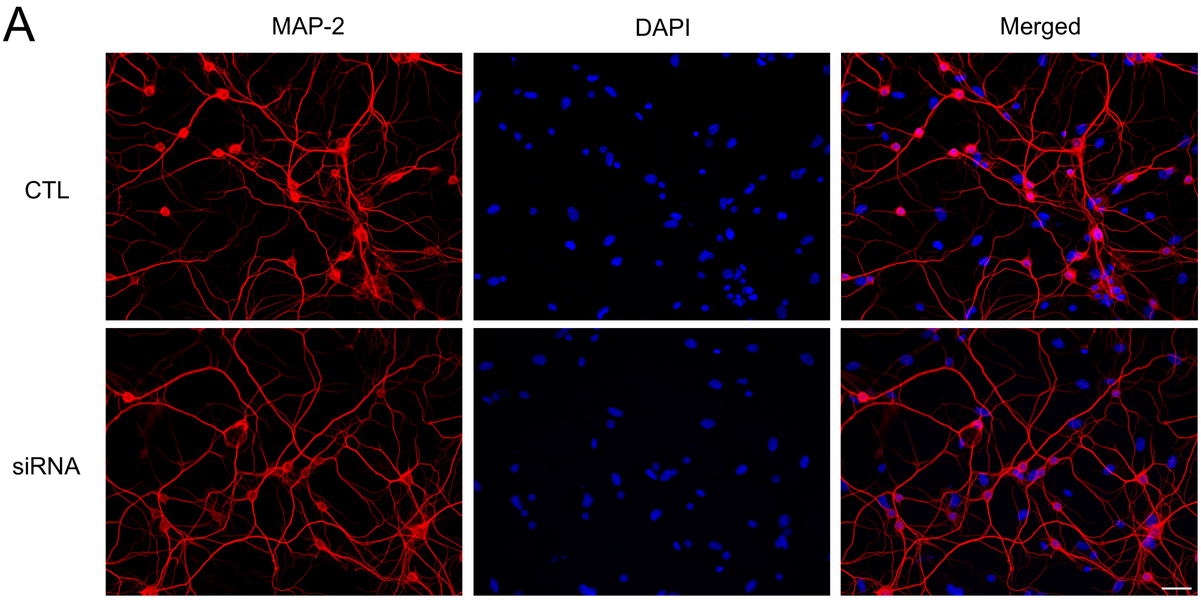

Supplementary Figure 3. A Representative full frame 20x images of control and siRNA cultures ICC stained with MAP-2. Scale bar represents 30 μm.

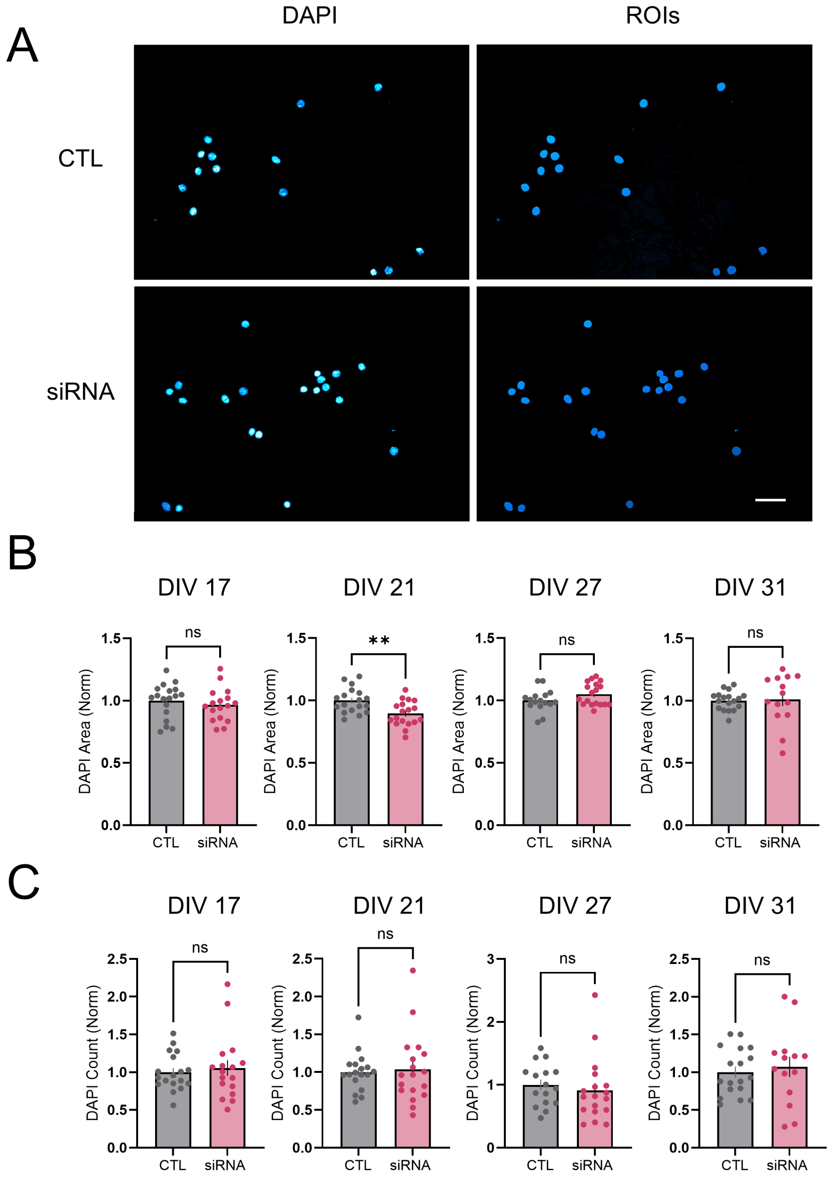

Supplementary Figure 4. **A** Left: Representative DAPI stained nuclei used to quantify total nuclear size and density. Right: Regions of interest (ROIs) generated using Imaris software based on thresholding DAPI signal to background intensity levels. Scale bar represents 50 µm. **B** Analysis of total nuclear size. **C** Analysis of total nuclear density. Data were assessed by unpaired t-tests and Mann-Whitney tests and are represented as mean ± SEM. ns: nonsignificant, ** $p < 0.01$.

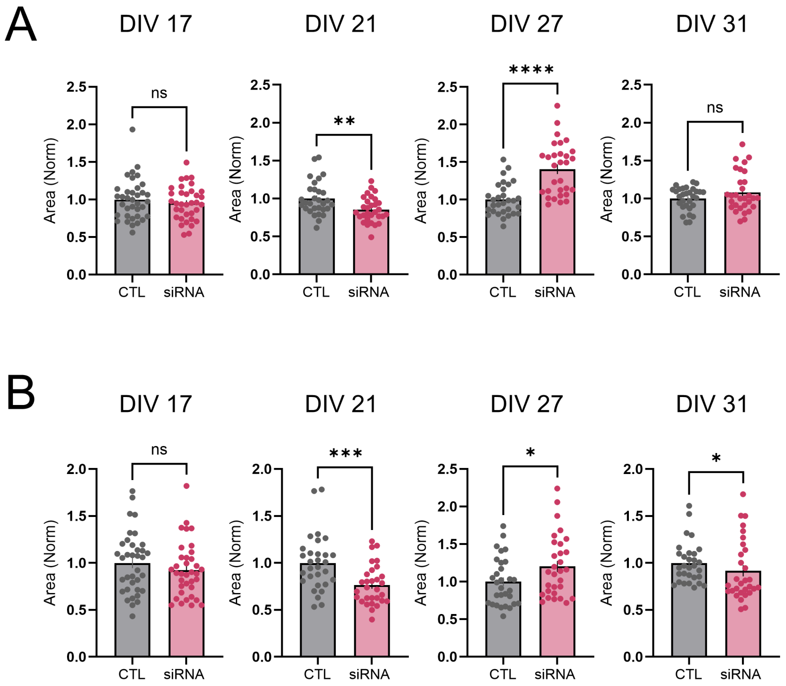

Supplementary Figure 5. **A** Analysis of nuclear size in MAP-2 positive neurons. **B** Analysis of cell body size in MAP-2 positive neurons. Data were assessed by Mann-Whitney tests and are represented as mean ± SEM. ns: nonsignificant, * $p < 0.05$, ** $p < 0.01$, *** $p < 0.001$, **** $p < 0.0001$.

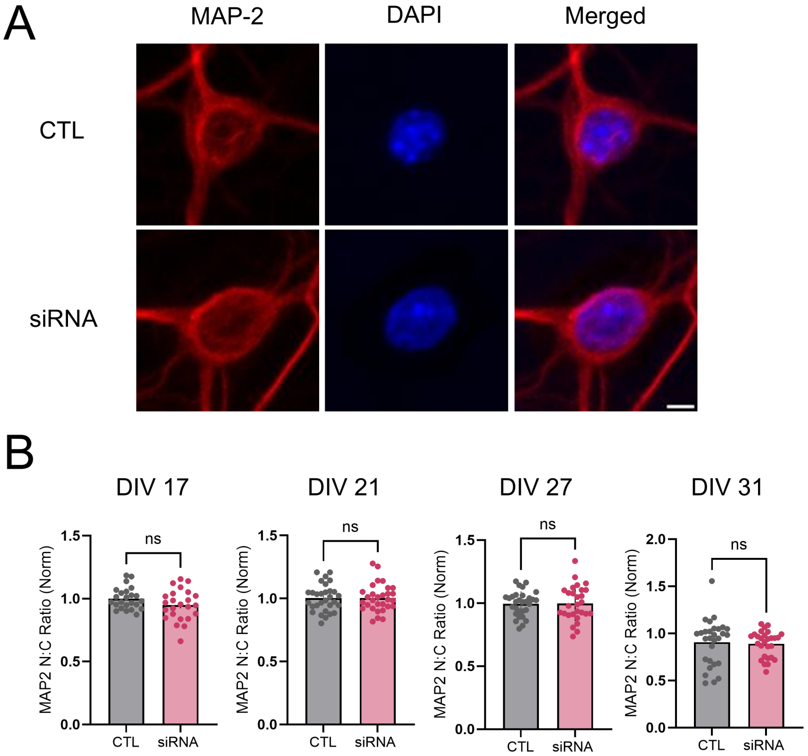

Supplementary Figure 6. **A** Representative MAP-2 (red) and DAPI (blue) stained neurons. Scale bar represents 3 μm. **B** Analysis of nuclear to cytosolic (N:C) MAP2 intensity ratios. Data were assessed by unpaired t-tests and Mann-Whitney tests and are represented as mean ± SEM. ns: nonsignificant.

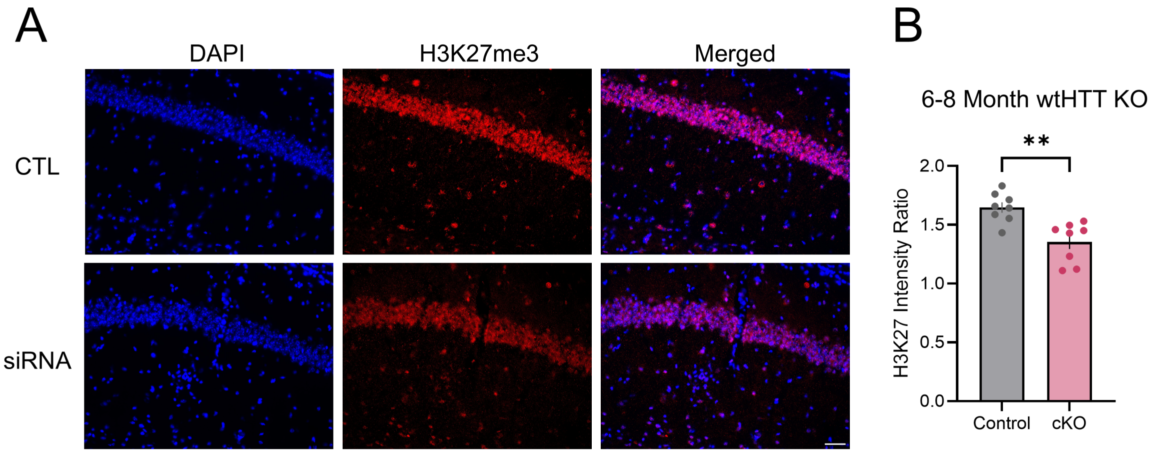

Supplementary Figure 7. **A** Representative examples of perfused slices immunostained with transcriptional repression marker, H3K27me3 and nuclear marker DAPI. Scale bar represents 30 µm. **B** Ratio of H3K27me3 nuclear ROI intensity to background in 6-8 month wtHTT KO mice. ROIs generated using FIJI thresholding protocol. Data were assessed by unpaired t-test and are represented as mean ± SEM. ** $p < 0.01$.
